# Pain sensitivity increases with sleep disturbance under predictable chronic mild stress in mice

**DOI:** 10.1038/s41598-021-93560-7

**Published:** 2021-07-09

**Authors:** Junhel Dalanon, Sachiko Chikahisa, Tetsuya Shiuchi, Noriyuki Shimizu, Parimal Chavan, Yoshitaka Suzuki, Kazuo Okura, Hiroyoshi Séi, Yoshizo Matsuka

**Affiliations:** 1 Department of Stomatognathic Function and Occlusal Reconstruction, Tokushima University Graduate School of Biomedical Sciences, Tokushima, Japan; 2 Department of Integrative Physiology, Tokushima University Graduate School of Biomedical Sciences, 3-18-15 Kuramoto-cho, Tokushima, 770-8504 Japan

**Keywords:** Stress and resilience, Sleep

## Abstract

Even though it has been well documented that stress can lead to the development of sleep disorders and the intensification of pain, their relationships have not been fully understood. The present study was aimed at investigating the effects of predictable chronic mild stress (PCMS) on sleep–wake states and pain threshold, using the PCMS rearing conditions of mesh wire (MW) and water (W) for 21 days. Exposure to PCMS decreased the amount of non-rapid eye movement (NREM) sleep during the dark phase. Moreover, the chronicity of PCMS decreased slow-wave activity (SWA) during NREM sleep in the MW and W groups in both the light and dark phases. Mechanical and aversively hot thermal hyperalgesia were more intensified in the PCMS groups than the control. Higher plasma corticosterone levels were seen in mice subjected to PCMS, whereas TNF-α expression was found higher in the hypothalamus in the W and the trigeminal ganglion in the MW group. The W group had higher expression levels of IL-6 in the thalamus as well. The PCMS paradigm decreased SWA and may have intensified mechanical and thermal hyperalgesia. The current study also suggests that rearing under PCMS may cause impaired sleep quality and heightened pain sensation to painful mechanical and aversively hot thermal stimuli.

Since the early 1980s, the chronic mild stress (CMS) model has been used to study the effects of stress^1^. The extensively used variant of CMS is the unpredictable CMS (UCMS). In this type of model, animals are subjected to unpredictable and variable stressors^1,2^. In contrast, the predictable CMS (PCMS) researches have just gained ground^3–6^. Previous PCMS experiments were directed at recovery from depression^3^, fear memory extinction^4^, resilience^5^, and hippocampal neurogenesis^6^.

A variety of uncontrollable stress paradigms have been used in past studies that resulted in the production of sleep disturbances or sleep alterations^7^. The amount of non-rapid eye movement (NREM) sleep may be altered by the stress paradigm^8^. Slow-wave activity (SWA) during NREM sleep is compellingly associated with sleep homeostasis and may be affected by stressful conditions^9^. SWA during NREM sleep has been shown to be imperative for physical and mental health maintenance, as well as brain plasticity and optimal recovery^10^. SWA in electroencephalography is a coordinated, oscillatory neocortical activity with an electrophysiological frequency from 0.5 to 4.0 Hz^11^ in rodents. After sleep deprivation, rebound sleep follows due to sleep homeostasis, and is exhibited by increased SWA during NREM sleep^12,13^.

In the study of Yasugaki et al. using water immersion and restraint stress, they found that UCMS resulted in depressive symptoms and increased rapid eye movement (REM) sleep in mice^14^. Experiments involving genetic rat models of depression using the Wistar-Kyoto^15^ and Flinders Sensitive Line showed elevated REM sleep as well^16^. Accumulated evidences indicate that various stresses have a profound effect on sleep, including NREM and REM sleep.

Unpredictable stress has also been reported to have a suppressive or facilitative influence on pain sensitivity. Chronic stress leads to hyperalgesia effects. In a previous study, it was reported that stressed rats experienced intensified mechanical allodynia^17^. Another study proposed that stress predisposes persistent thermal hyperalgesia in mice^18^. However, few animal studies have examined the link between stress and pain, and the mechanism remains unknown. High comorbidity linking sleep problems and chronic pain has been reported by several studies. Restless leg syndrome, obstructive sleep apnea, and insomnia were most common in chronic pain patients with a prevalence of sleep problems, according to a recent meta-analysis^19^. Despite the apparent link between chronic pain and sleep disorders, it is still not yet fully understood to what extent one affects the other.

Cytokines are candidate substances for the mechanism that links stress, sleep, and pain. Proinflammatory cytokines’ tumor necrosis factor alpha (TNF-α) and interleukin 6 (IL-6) are commonly implicated in studies involving inflammation, thus these were explored in the current study. Sleep deprivation experiments have shown that TNF-α and IL-6 are possible intermediaries of extreme sleepiness at daytime^20^. Although the mechanism is unclear, these cytokines are associated with sleep disturbance^21^ and hyperalgesia^22^ under CMS.

Primarily, this study aimed to examine whether PCMS can cause sleep disturbances and increase pain threshold, and understand how PCMS can affect sleep and pain. Therefore, using continuous multiple PCMS rearing conditions for 21 days, the effects of predictable stress on the changes in sleep–wake stages and the sensation of pain were examined. Following the establishment of the different rearing environments that led to stress, investigations were done to find out if: (i) changes in sleep amount (wake, NREM sleep, and REM sleep) and SWA during NREM sleep were produced; (ii) mechanical and thermal pain sensations were aggravated; (iii) neurobiological changes in the mRNA expression levels of cytokines in different brain regions, corticosterone in trunk blood plasma occured.

## Results

### PCMS leads to stunted weight but greater food intake in the water group

**Figure 1:**
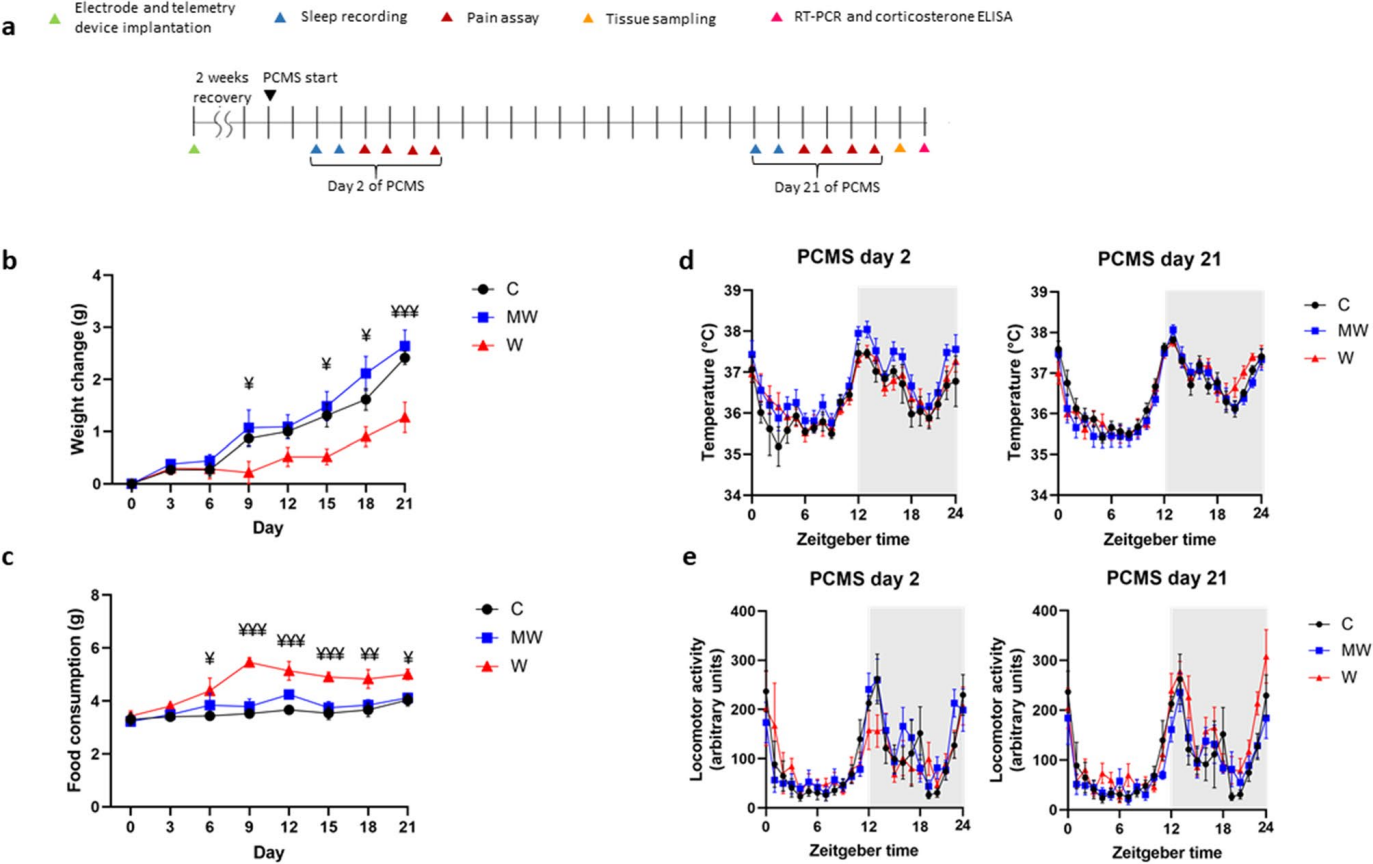
Weight changes, food consumption, core body temperature, and locomotor activity. The experiment timeline showing sleep and pain tests during predictable chronic mild stress (PCMS) (**a**). Significant weight decrease was seen in the water (W) group on days 9, 15, 18, and 21 of the PCMS timeline compared to the control (C) group (**b**). Food consumption increased for the W group on days 6, 9, 12, 15, 18, and 21 (**c**). No changes in terms of core body temperature were observed (**d**). There were no significant changes in locomotor activity as well (**e**). N = 6–7 per group; ¥*p* < 0.05, ¥¥*p* < 0.01, ¥¥¥*p* < 0.001 (W vs C); two-way repeated-measures ANOVA followed by Dunnett’s multiple comparison test. Data are presented as means ± SEM.

A control (C) cohort reared in regular sawdust was compared to mice reared on mesh wire with regular sawdust (MW) and those reared on mesh wire with water (2 mm below the mesh wire) (W) group (Fig. 1a and Supplemental Fig. S1d). Differences in weight changes were seen across the PCMS timeline (Fig. 1b, F_(14, 118)_ = 3.691, *P* < 0.001). The curbed weight was particularly observed in the W group on days 9 (*P* = 0.046), 15 (*P* = 0.012), 18 (*P* = 0.026), and 21 (*P* < 0.001) compared to the C group. Changes across the PCMS timeline in terms of food consumption yielded some significance (Fig. 1c, F_(14, 114)_ = 1.941, *P* = 0.029), but it shows that the W group developed greater appetite on days 6 (*P* = 0.014), 9 (*P* < 0.001), 12 (*P* < 0.001), 15 (*P* < 0.001), 18 (*P* = 0.002), and 21 (*P* = 0.013) in contrast with the C group.

### Core body temperature (CBT) and locomotor activity (LA) were unaffected by PCMS

The application of predictable stress did not affect CBT on day 2 (Fig. 1d, F_(46, 414)_ = 0.6233, *P* = 0.975) and day 21 (Fig. 1d, F_(46, 414)_ = 1.163, *P* = 0.224) of the PCMS timeline. Analogously, the LA during day 2 (Fig. 1e, F_(46, 414)_ = 1.056, *P* = 0.379) and day 21 (Fig. 1e, F_(46, 414)_ = 0.9231, *P* = 0.618) were not affected by the predictability of stress conditions in the MW and W groups compared with the C group.

### PCMS decreased the SWA during NREM sleep

**Figure 2:**
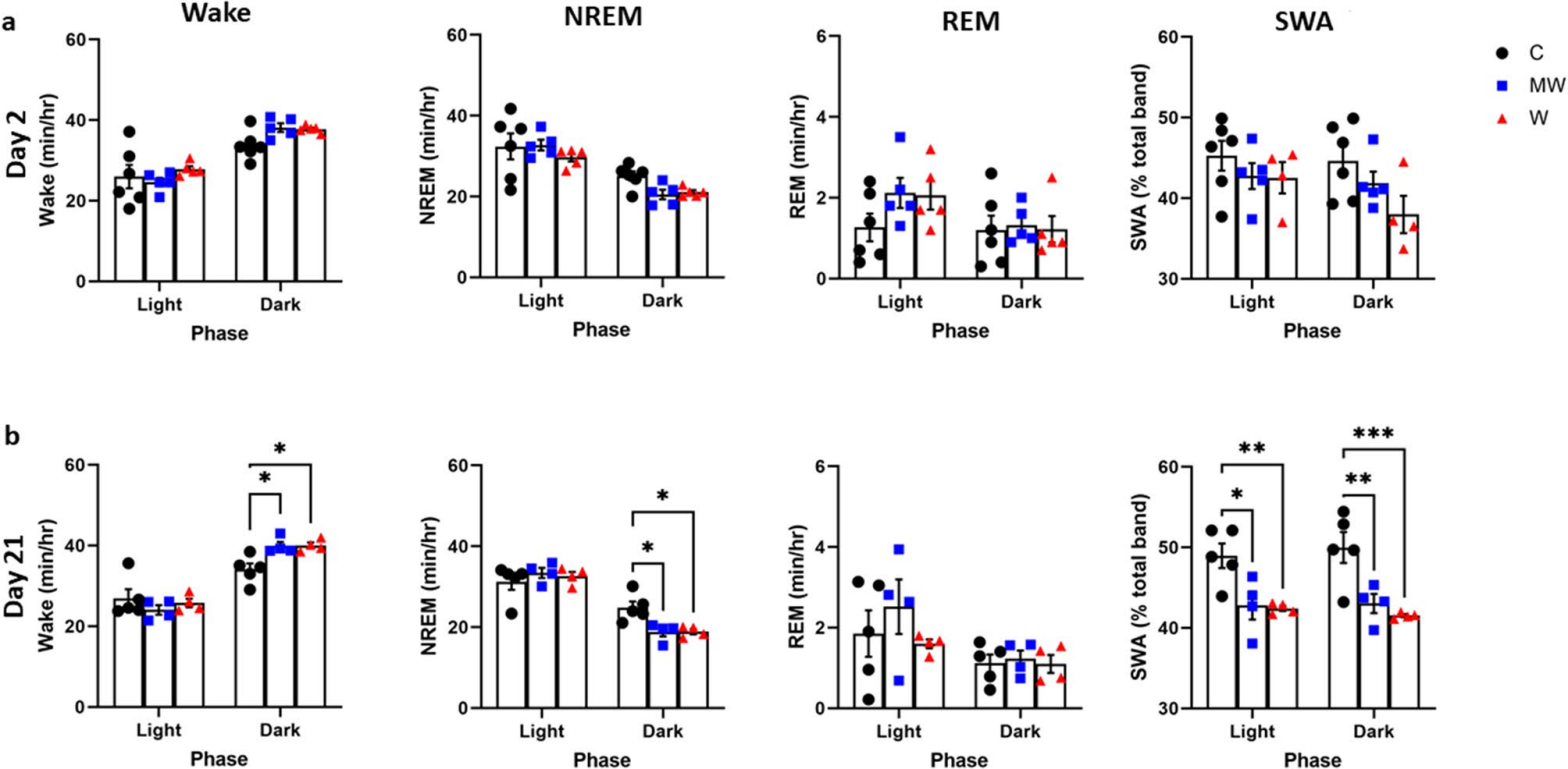
Sleep–wake stages during days 2 and 21 of the PCMS timeline per phase. There were no noticeable disparities in the amount of wake, rapid eye movement (REM) sleep, and slow-wave activity (SWA) during non-rapid eye movement (NREM) sleep on day 2 of predictable chronic mild stress (PCMS). The amount of NREM sleep tends to decrease during the dark phase (**a**). On day 21 of PCMS, increase in the amount of wake, while decrease in the amount of NREM sleep and SWA during NREM sleep were observed. A W group mouse containing artificial noise was excluded from SWA analysis on day 2 (**b**). N = 4–6 per group; *p < 0.05; **p < 0.01; two-way ordinary ANOVA followed by Dunnett’s multiple comparison test. Data are presented as means ± SEM.

On day 2 of PCMS, there were no changes in the amount of wake, REM sleep, and SWA during NREM sleep across the phases, while the amount of NREM sleep tended to decrease in MW and W groups (Fig. 2a). This observation was similar when the values across zeitgeber time (ZT) were compared (Supplemental Fig. S2a). The duration of episodes in the dark phase of NREM sleep decreased in MW (*P* = 0.040) and W (*P* = 0.011) groups (Supplemental Fig. S3a, F_(2, 13)_ = 5.898, *P* = 0.015). On day 21 of PCMS, the MW (*P* = 0.013) and W (*P* = 0.012) groups compared to the C group manifested an overall increase in the amount of wake during the dark phase (Fig. 2b, F_(2, 11)_ = 5.192, *P* = 0.026). In conjunction, the amount of NREM sleep of MW (*P* = 0.008) and W (*P* = 0.008) groups significantly decreased compared with that of C group in the same phase (Fig. 2b, F_(2, 11)_ = 5.331, *P* = 0.024).The episode number of NREM sleep in the dark phase tended to decrease in the MW and W groups (Supplemental Fig. S3b, F_(2, 10)_ = 4.621, *P* = 0.038).

In contrast, on day 2, SWA during NREM sleep in the dark phase tended to decrease in W group (Fig. 2a) and the EEG power spectra in the SWA frequency band during NREM sleep also decreased in the dark phase in W group only (Supplemental Fig. S4a). Correspondingly, on day 21 of PCMS, both SWA (Fig. 2b) and EEG spectra (Supplemental Fig. S4a) during NREM sleep significantly decreased in both light and dark phases. There was no significant effect on EEG spectra during REM sleep (Supplemental Fig. S4b).

### Heightened mechanical pain sensation in the water group

**Figure 3:**
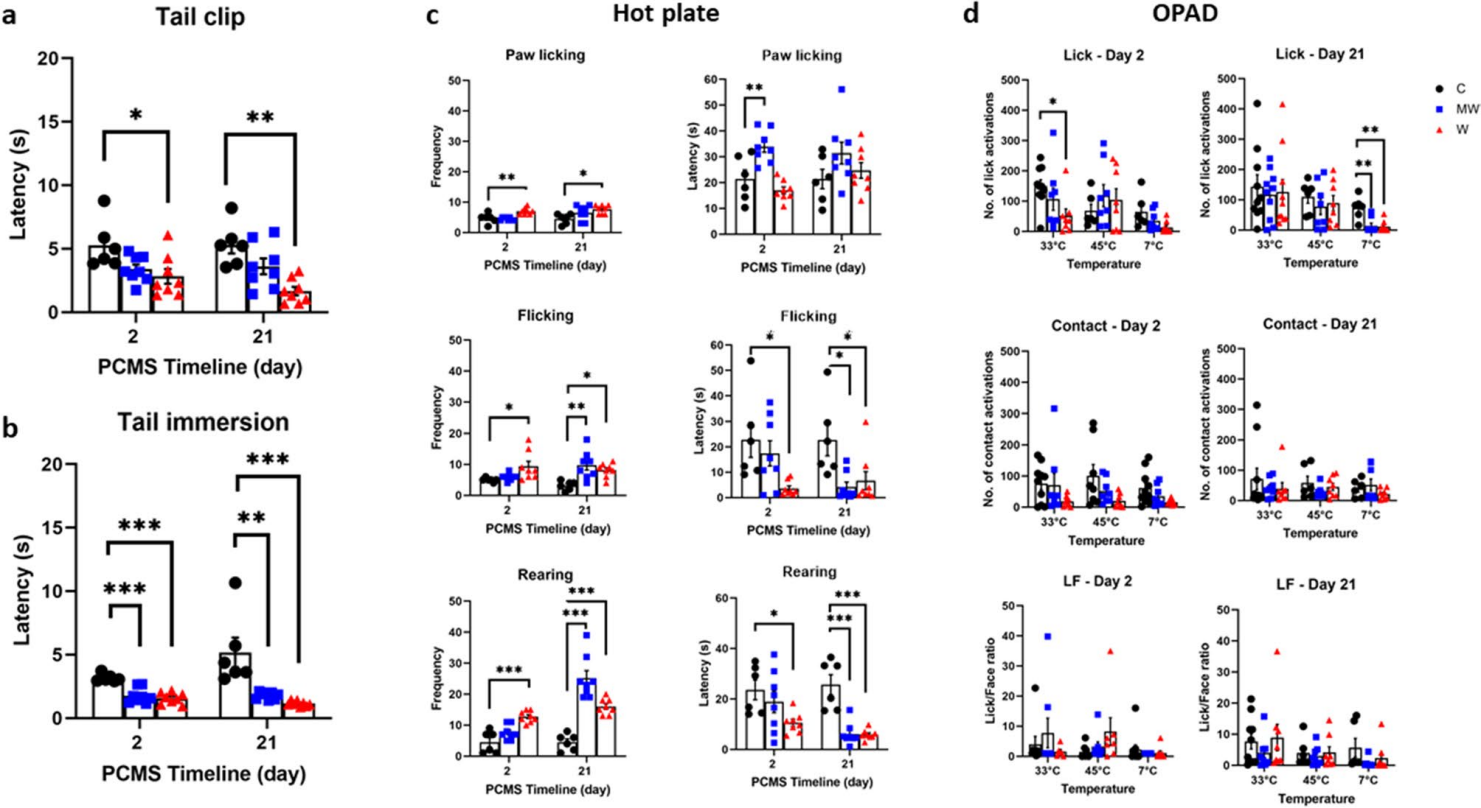
Quantitative sensory testing for pain. The water (W) group had lower mechanical pain threshold on days 2 and 21 of the predictable chronic mild stress (PCMS) based on the results of the tail clip test (**a**). The mesh wire (MW) and W groups were more sensitive to thermal pain on both day 2 and day 21 of the PCMS according to the faster latency in the tail immersion test (**b**). The hot plate test frequency on day 2 and day 21 revealed greater pain for the MW and W groups (**c**). There was noticeable increase in orofacial pain sensations brought about by decrease in the number of licks in the W group at 33 °C on day 2 and in the MW and W groups at 7 °C on day 21. The orofacial pain sensitivity was assessed using the orofacial pain assessment device (OPAD) (**d**). N = 6–8 per group (tail clip, tail immersion, and hot plate); N = 8–10 per group (OPAD); *p < 0.05, **p < 0.01, ***p < 0.001; one-way ANOVA followed by Dunnett’s or Tukey’s multiple comparison test. Data are presented as means ± SEM.

Mechanical hyperalgesia was observed on day 2 (Fig. 3a, F_(2, 19)_ = 4.590, *P* = 0.024) in the W group (*P* = 0.015), and day 21 (Fig. 3a, F_(2, 19)_ = 10.44, *P* < 0.001) on the same W group (*P* < 0.001), subjected to the tail clip test.

### Faster reaction time to aversive thermal stimulus on PCMS

Increased sensitivity to aversive hot temperature was also observed amongst the PCMS groups in the tail immersion test on day 2 (Fig. 3b, F_(2, 19)_ = 21.69, *P* < 0.001) and day 21 (F_(2, 19)_ = 14.30, *P* < 0.001). All of the mice under the different PCMS conditions of MW (*P* < 0.001) and W (*P* < 0.001) experienced more pain induced by hot temperature on day 2 than the C group. On day 21, the MW (*P* < 0.001) and W (*P* < 0.001) compared to the C group had aggravated thermal pain as well.

### PCMS induced faster latency and more frequent reaction to the hot plate test

Significantly greater pain were experienced by the PCMS groups in terms of frequency in paw licking (Fig. 3c, F_(2, 19)_ = 12.26, *P* < 0.001), flicking (Fig. 3c, F_(2, 19)_ = 4.615, *P* = 0.023), and rearing frequency (Fig. 3c, F_(2, 19)_ = 18.73, *P* < 0.001) during the hot plate test on day 2. The same can be observed on day 21 in terms of paw licking (Fig. 3c, F_(2, 19)_ = 4.617, *P* = 0.023), flicking (Fig. 3c, F_(2, 19)_ = 7.996, *P* = 0.003), and rearing latency (Fig. 3c, F_(2, 19)_ = 33.13, *P* < 0.001). On day 2, the W group showed more paw licking (*P* = 0.002), flicking (*P* = 0.022), and rearing (*P* < 0.001). On day 21, the W group similarly expressed greater paw licking (*P* = 0.013), flicking (*P* = 0.015), and rearing (*P* < 0.001). The MW group also showed more frequency of flicking (*P* = 0.002) and rearing (*P* < 0.001) on day 21.

Hot plate reaction latency on day 2 also showed significant changes in terms of paw licking (Fig. 3c, F_(2, 19)_ = 15.52, *P* < 0.001), flicking (Fig. 3c, F_(2, 19)_ = 4.714, *P* = 0.022), and rearing frequency (Fig. 3c, F_(2, 19)_ = 3.675, *P* = 0.045). Similar observations for flicking (Fig. 3c, F_(2, 19)_ = 6.059, *P* = 0.009) and rearing frequency (Fig. 3c, F_(2, 19)_ = 25.75, *P* < 0.001) occurred on day 21 of PCMS. On day 2, the W group showed faster flicking (*P* = 0.017) and rearing (*P* = 0.003), while the MW group showed slower paw licking (*P* = 0.003). On day 21, the latency reaction shown by the W group was faster in terms of flicking (*P* = 0.019) and rearing (*P* < 0.001). Similarly, the MW group showed faster flicking (*P* = 0.007) and rearing (*P* < 0.001) as well.

### PCMS promotes orofacial pain hypersensitivity with aversive cold stimulus

According to the orofacial pain assessment device (OPAD) results, there was a noticeable increase in orofacial pain sensations brought about by a decrease in the number of licks (Fig. 3d, F_(2, 22)_ = 3.674, *P* = 0.042) in the W group (*P* = 0.024) at 33 °C on day 2. Decreases in licks (Fig. 3d, F_(2, 19)_ = 11.03, *P* < 0.001) were also observed in the MW (*P* = 0.001) and W (*P* < 0.001) groups at 7 °C on day 21.

### Upregulation of TNF-α in the hypothalamus and trigeminal ganglion, IL-6 in the thalamus after PCMS

**Figure 4:**
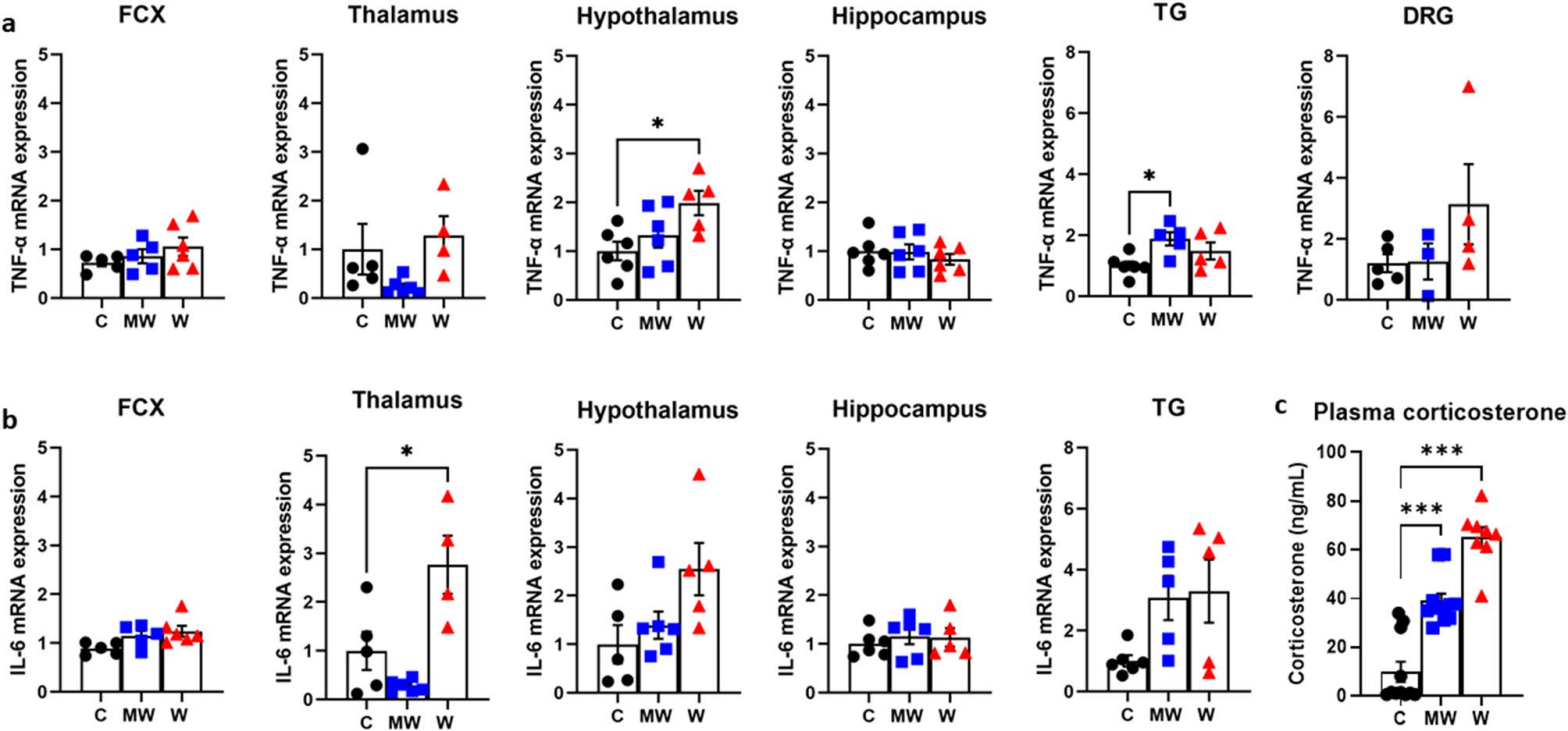
Comparison of RT-PCR results of cytokine expression. In terms of tumor necrosis factor alpha (TNF-α) expression, no significant changes were seen in the frontal cortex (FCX), thalamus, hippocampus, and dorsal root ganglion (DRG). However, significant increases in the hypothalamus were observed in the water (W) group and the trigeminal ganglion (TG) in the mesh wire (MW) compared to the control (C) group (**a**). Higher expressions of interleukin-6 (IL-6) were found in the thalamus of the W group only (**b**). Plasma corticosterone levels were higher across all predictable chronic mild stress (PCMS) groups than the control (**c**). N = 4–6 per group (RT-PCR); N = 8–11 per group (Corticosterone); *p < 0.05, ***p < 0.001; one-way ordinary ANOVA followed by Dunnett’s multiple comparison test. Data are presented as means ± SEM.

In terms of TNF-α expression, no significant changes were seen in the frontal cortex (FCX) (*P* = 0.319), thalamus (*P* = 0.124), hippocampus (*P* = 0.637), and dorsal root ganglion (DRG) (*P* = 0.224). However, significant increases in the hypothalamus (Fig. 4a, F_(2, 14)_ = 4.499, *P* = 0.031) were observed in the W group (*P* = 0.019) and the trigeminal ganglion (TG) (Fig. 4a, F_(2, 13)_ = 4.495, *P* = 0.033) in the MW (*P* = 0.020). Higher expressions of IL-6 were found in the thalamus (Fig. 4b, F_(2, 12)_ = 12.34, *P* = 0.001) of the W group (*P* = 0.011) only. In addition, the data suggest that increases in hypothalamic IL-6 expression (*P* = 0.052) and FCX (*P* = 0.054) in the W group may be potentially significant.

### Increased levels of plasma corticosterone under PCMS

The plasma corticosterone levels of the PCMS groups markedly deviated on day 21 of the PCMS timeline (Fig. 4c, F_(2, 27)_ = 50.73, *P* < 0.001). The MW (38.99 ± 3.01 ng/mL, *P* < 0.001) and W group (65.24 ± 4.11 ng/mL, *P* < 0.001) all increased plasma corticosterone level compared to the C group (9.83 ± 4.16 ng/mL).

### SWA during NREM sleep changed with pain sensation in mice under PCMS

**Figure 5:**
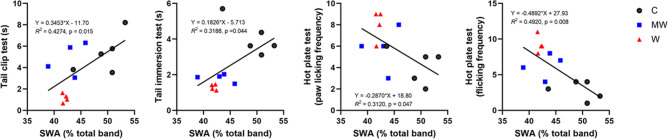
Correlation between sleep and pain on day 21 of PCMS. Scatter plot showing a positive correlation between slow-wave activity (SWA) during non-rapid eye movement sleep and reaction time to tail clip test, as well as between SWA and tail immersion test. Similarly, SWA shares a negative correlation between the hot plate test paw licking frequency and flicking frequency in the control (C), mesh wire (MW), and water (W) groups. SWA values used are the average values for 24 h. N = 4–5 per group; Pearson correlation test.

The results on day 2 convey no significant relationships between sleep and pain. Conversely, on day 21, SWA during NREM sleep was found to have significant positive correlations with the reaction time in tail clip test (Fig. 5, R^2^ = 0.4274, *P* = 0.015) and in tail immersion test (Fig. 5, R^2^ = 0.3188, *P* = 0.044). Comparably, SWA during NREM sleep had significant negative correlations with frequency of paw licking (Fig. 5, R^2^ = 0.3120, *P* = 0.047) and flicking (Fig. 5, R^2^ = 0.4920, *P* = 0.008) in the hot plate test.

## Discussion

The present study provides evidence supporting the use of PCMS as a promising animal model in the study of sleep disturbances and pain sensitivity. The hypothalamic–pituitary–adrenal (HPA) axis activation is one of the most constant neurobiological features of stress models^23^. The PCMS cohorts in the current study had significantly elevated levels of plasma corticosterone (Fig. 4c). This result suggests that PCMS activates the HPA axis.

The increase in corticosterone indicates an activation of the HPA axis and may accompany weight changes. The results of previous UCMS studies reported that stress can significantly lower body weight^24^. In addition, the bodyweight of mice under restraint stress for 15 days remained lower despite the improvement in food intake^25^. The results in the present study showed a decreased bodyweight gain of mice in the W group despite the increase in food intake. Considering previous research mentioned above, these are thought to be due to the activation of HPA axis under PCMS. In addition to the action of the HPA axis due to stress, this increase in food consumption but inhibited weight gain in the W group, may be due to impaired sleep quality. Pathologic disturbance in sleep homeostasis can stimulate the appetite, but it could also trigger energy expenditure increase that could stunt weight gain. In other words, the increase in energy supply resulting from increased food consumption could be offset by increased energy demand for extended awake time. On the other hand, mice of MW group experienced sleep disturbance as well as W group but showed similar weight gains and food consumption as the C group. These results may indicate that the intensity of stress in MW group was milder than W group.

The exposure to PCMS caused the mice in MW and W group to lack NREM sleep during the dark phase on day 21 (Fig. 2b) but not on day 2 (Fig. 2a). As PCMS is a depression model, this datum is supported by earlier studies showing diminished NREM sleep in patients with depression^14,26^. The current study suggests that REM sleep is not affected by PCMS. Prior experiments on UCMS insinuate that unpredictable stress can result in depressive symptoms, increase REM sleep^14^, and shortened latency to REM sleep^26^. These differences could be due to varying types of models, intensity, and length of time these stresses were applied.

Our PCMS protocol managed to diminish SWA during NREM sleep in the MW and/or W groups (Fig. 2b). SWA during NREM sleep is the soundest described biological indicator of sleep homeostasis^27^. Previously, it was reported that attenuation of homeostatic sleep management by SWA during NREM sleep may be due to stress^28^. Similarly, our PCMS protocol also caused impairment of sleep homeostasis, and mice in MW and W groups lost deep sleep. Regarding pain, an increase in pain sensation in W group was already observed from day 2 on tail clip test (Fig. 3a) and almost all parameters of the hot plate test (Fig. 3c). These results suggest that change in pain sensation occurred earlier in W group than in MW group. On the other hand, in the tail immersion test using hot water, WM group and W group responded in almost the same way, but this would be because W group is familiar with water and could not detect the difference in pain sensitivity with MW. Since a decrease in SWA during NREM sleep seen in the power spectra was also observed only in W group on day 2 of PCMS (Supplemental Fig. S4a), change in pain sensation seems to occur in parallel with changes in sleep quality (SWA). Furthermore, SWA has significant correlation with the sensations of mechanical hyperalgesia and thermal hyperalgesia when predictable stress was perpetuated until day 21 (Fig. 5), the results may indicate that the decrease in SWA during NREM sleep caused by PCMS have contributed to the increased pain sensations of the mice. This may indicate the importance of sleep quality on the pain control.

Chronic stress is a comorbidity of chronic pain. The various rearing models yielded variable results, where the W group reported the most pain sensitivity during day 21 of the PCMS timeline (Fig. 3). The cytokines, immune system signaling molecules, may elucidate the relationship between these conditions^29^. In the current study, the results of the MW and W group show significantly increased TNF-α expressions (Fig. 4a), while IL-6 was also increased in the thalamus (Fig. 4b). According to a CMS study, the expression of cytokines, including TNF-α and IL-6, were elevated in various regions of the brain during inflammation^30^. It has been known that IL-6 is implicated in the reaction of neurons during nerve injury^31^. The role of TNF-α in some pain models has also been well-established. Previous experiments have shown that injections of TNF-α may produce thermal^32^ and mechanical^33^ pain. These stress-related studies support the findings of the current study that inflammatory cytokines, like TNF-α and IL-6, may be implicated in the exacerbation of pain caused by the inflammatory responses during PCMS. Some studies report that sleep inhibition may increase cytokine expression in the brain^34^. In this study, TNF-α and IL-6 were elevated in the brain of sleep-inhibited groups (MW and W). Hyperalgesia was also observed in these groups. These point to the fact that sleep disturbance caused by PCMS may have induced hyperalgesia through elevated brain cytokines.

While this study gives a baseline for the effect of PCMS on sleep and pain, there are various facets in the mechanism that still need to be understood. For instance, there exist several differences in the elicitations of pain, sleep, and stress in terms of behavior and biochemical markers. Another interesting viewpoint would be the comparison of PCMS to UCMS. Moreover, gender differences may yield noteworthy results. Anti-inflammatory cytokines, injection of exogenous cytokines, and the use of knockout mice may also produce important results. These are some of the limitations of this study and these experiment modifications can elucidate further the relationship between sleep and pain under the PCMS paradigm. In addition, the acquisition of continuous sleep data between day 2 and day 21 may further elucidate the relationship between sleep and pain under predictable stress.

## Materials and methods

### Animals

Eight-week-old, 24–26 g, male C57BL/6J mice (Japan SLC, Shizuoka, Japan) were used in all of the experiments (Supplemental Fig. S1a). Mice were housed in a temperature (23 ± 1 °C) and humidity-controlled (40%) vivarium with a 12-h light–dark (L/D) cycle (lights on at 8:00 a.m.). Regular food chow and water were provided ad libitum. Ethics approval was sought and given by the Animal Study Committee of Tokushima University (License No. T30-30). All experimental procedures were done under the Guidelines for the Care and Use of Animals approved by the Council of the Physiological Society of Japan, and the International Association for the Study of Pain Guidelines for the Use of Animals in Research. Adherence to transparent reporting was followed through the updated Animal Research: Reporting of In Vivo Experiments (ARRIVE 2.0) guidelines. All efforts were exhaustively done to reduce animal suffering and to lessen the number of animals used.

### Stereotactic implantation of electrodes for sleep recording

Intraperitoneal injection of a ketamine (100 mg/kg) and xylazine (25 mg/kg) concoction was done to anesthetize the mice. A stereotactic device was used for stabilization and reference-point measurements for electrode placement. Epidural implantation of two anterior (1.5 mm rostral and 1.5 lateral) and two posterior (2.5 mm caudal and 2.5 lateral) EEG (electroencephalogram) miniature screw electrodes were done in the skull with the bregma as reference point. For EMG (electromyogram) recording, bilateral attachment of Teflon-coated stainless-steel wires in the trapezius muscle was performed (Supplemental Fig. S1b).

### Peritoneal attachment of telemetry device for locomotion and core body temperature measurement

An implantable telemetry device (Nano tag, Kissei Comtec, Matsumoto, Japan) was embedded in the left peritoneal cavity. It uses a thermal and three-axis accelerometer to measure the mice’s activity and CBT (Supplemental Fig. S1c).

### Predictable chronic mild stress rearing conditions

After a recovery period of fourteen days, mice were housed individually in individual cages (136 × 208 × 115 mm) and placed under different rearing conditions. The experimental groups were segregated into a C group and the PCMS models of MW, and W. The C mice were reared in bedding filled with regular sawdust; MW was reared on wire net on top of regular sawdust; the W was placed in a cage with water, 2 mm below the wire net for 21 days (Supplemental Fig. S1d).

### Polysomnographic recording of sleep–wake stages and data analysis

Sleep recordings were done in a soundproof recording chamber, where the subjects were transferred to plastic cages (200 × 240 × 300 mm) and were habituated for two days. The EEG/EMG electrode assembly was connected to a computer-assisted data recording infrastructure (Vital Recorder, Kissei Comtec, Matsumoto, Japan). Polysomnographic recordings of EEG/EMG, CBT, and LA were retrieved during baseline (Day 0), second day of stress (Day 2), and the twenty-first day of PCMS (Day 21). Each recording session started at ZT 0 and lasted for 48 h, under a 12-h L/D cycle, under a controlled environment, and ad libitum feeding. Via visual valuation of the acquired data on the polysomnographic activity using SleepSign analysis program (Kissei Comtec, Matsumoto, Japan), asynchronous sleep scoring was done. The vigilance states of wake, NREM sleep, and REM sleep were determined through spectrum and pattern analysis using a fast Fourier transform (FFT) algorithm. Manual classification of vigilance states per epoch, as wake, NREM sleep, or REM sleep per 10-s epoch based on EEG patterns was done. Epochs containing artificial noise were excluded from FFT analysis. Constant EEG theta waves and absolute repression of EMG are characteristics of REM sleep. In contrast, high-voltage and slow EEG with low-voltage EMG activity are indicative of NREM sleep. The delta and theta frequency bands of EEG were set at 0.5–4.0 Hz (SWA) and 4.5–10.5 Hz, correspondingly (Supplemental Fig. S1e).

### Quantitative sensory testing for pain threshold

Pain threshold against mechanical hyperalgesia was measured using the tail clip test, while thermal hyperalgesia was measured by tail immersion test and hot plate test. The OPAD was used to ascertain the orofacial pain sensations at aversive hot and cold temperatures. Tests were performed on days 2 and 21. Every behavioral test was done from 8:00 a.m. to 3:00 p.m. during the light phase (Supplemental Fig. S1f–i).

### Tail clip test

The tail clip test was done using a modified version of Takagi et al.’s protocol^35^. A metal clip was applied to the base of the tail. The reaction time was determined from when the clip is attached until the attempted dislodgement by the subject. Each session was done in triplicates at five-minute intervals and the average reaction time was recorded. A ten-second cut-off time was observed to avoid permanent nerve damage. Unresponsive mice were excluded from the computation (Supplemental Fig. S1f).

### Tail immersion test

Adopting a modified version of Adeyemi et al.’s tail immersion test, a hot water bath (55.0 ± 0.5 °C) was used where the lower 5 cm part of the tail was dipped in^36^. The reaction time is calculated as the average in triplicates at five-minute intervals of the time it took in seconds for the subject to withdraw the tail out of the water (Supplemental Fig. S1g).

### Hot plate test

As a test for thermal sensitivity unaffected by stress-induced analgesia linked with restraint, the hot plate test was used. Each mouse was gradually positioned at the center of the plate (55.0 ± 0.5 °C) under an enclosure. The frequency and initial reaction time for the paw-licking, jumping, flicking, and rearing was recorded as responses^37^. A sixty-second cut-off time was observed to avoid any potential nerve damage (Supplemental Fig. S1h).

### Orofacial pain assessment device

The OPAD, a high throughput operant assay, was used to detect orofacial pain. The buccal hair of the subjects was shaved 1–2 days before testing and food-fasted (15 ± 1 h) before each session to ensure behavior recording accuracy. Three sessions of training per week at non-aversive temperatures (33–37 °C), for mice to lick at 600–1000 times, was done before the actual recording sessions. The accompanying software, ANY-maze (Stoelting Co., Wood Dale, IL), was calibrated to run the session for 18 min with ramping normal (33 °C), aversive hot (45 °C), and aversive cold (7 °C) temperatures. The subject was placed in a Plexiglas cage with metal flooring grate with Peltier thermodes that come into contact before it can feed on the reward bottle. The number of lick activations and contact activations were automatically recorded as measures of behavioral pain outcomes. The lick to face ratio was computed by dividing the number of licks by the number of contacts (Supplemental Fig. S1i). A decline in the number of lick, contacts, and lick-face ratio indicates an increase in orofacial pain sensation^38^.

### Real-time RT-PCR analysis

After the last day of the PCMS protocol, mice were euthanized by rapid cervical dislocation. Instantaneously after decapitation, tissues used for molecular analysis were snap-frozen in liquid nitrogen and kept at  − 80 °C. Adhering to Takara’s RNA isolation protocol (RNAisoPlus; Takara Bio, Shiga, Japan), total RNA of FCX, thalamus, hypothalamus, hippocampus, TG, and DRG were isolated. A high-capacity cDNA transcription kit (Applied Biosystems, Foster, CA, USA) was used to generate cDNA from each tissue RNA sample. Gene-specific and predesigned TaqMan primer sets and probes (Applied Biosystems, Foster City, CA) were used to ascertain TNF-α and IL-6 gene expression. The Applied Biosystems 7900HT real-time RT-PCR system and TaqMan Universal PCR Master Mix (Roche Applied Science, Mannheim, Germany) were used to perform real-time RT-PCR corresponding to the guidelines of the manufacturer. Normalization of the values to those of housekeeping gene beta-actin was done for endogenous quality control. Attributable to the small size of some tissues or low concentration of mRNA, the RT-PCR results in some cases yielded no data value. In these instances, the values cannot be considered for inclusion in the final statistical analysis.

### Measurement of plasma corticosterone levels

Immediately after decapitation, trunk blood was collected from each group for the measurement of corticosterone levels. To separate the plasma, whole blood samples containing EDTA–2Na (1 mg/ml) were centrifuged (4 °C, 4000 rpm, 15 min). Using an enzyme immunoassay kit (Yanaihara Institute Inc., Fujinomiya, Japan) and following the manufacturer’s instructions, plasma corticosterone level was measured.

### Statistical analyses

In comparing weight change, food consumption, LA, CBT, wake-sleep stages, and SWA during NREM sleep per ZT, and EEG power spectra a two-way repeated-measures ANOVA was used. A two-way ordinary ANOVA was used for the comparison of sleep–wake stages per phase, and episode duration and number. While a one-way ordinary ANOVA was used to evaluate differences in RT-PCR, corticosterone enzyme immunosorbent assay, quantitative sensory testing for pain, and OPAD results. Correlational analyses between SWA during NREM sleep and pain test values were done using Pearson correlation coefficients. Dunnett’s or Tukey’s multiple comparison test was used for posthoc analysis when needed. Gaussian distribution was evaluated using the Shapiro–Wilk normality test. Unless otherwise specified, data are reported as mean ± standard error of the mean (SEM). All statistical tests were performed using GraphPad Prism 9 for Windows (GraphPad Software, La Jolla California USA).

## Supplementary Information

Supplementary Information.

## Data Availability

Data analyzed or generated in this study are included in this manuscript. Datasets not included are available from the corresponding author upon reasonable request.
