# Supplementary material for: Pain sensitivity increases with sleep disturbance under predictable chronic mild stress in mice
Source: Sci Rep. 2021 Jul 9;11:14231. doi: 10.1038/s41598-021-93560-7 (PMC8271003; doi:10.1038/s41598-021-93560-7)
Supplement: Supplementary file 1 — Supplementary Information. [file 41598_2021_93560_MOESM1_ESM.docx]

**Pain sensitivity increases with sleep disturbance under predictable chronic mild stress in mice**

Junhel Dalanon ^1^, Sachiko Chikahisa ^2 *^, Tetsuya Shiuchi ^2^, Noriyuki Shimizu ^2^,

Parimal Chavan ^1^, Yoshitaka Suzuki ^1^, Kazuo Okura ^1^, Hiroyoshi Séi ^2^, Yoshizo Matsuka ^1^

^1^ Department of Stomatognathic Function and Occlusal Reconstruction, Tokushima University Graduate School of Biomedical Sciences, Tokushima City, Japan

^2^ Department of Integrative Physiology, Tokushima University Graduate School of Biomedical Sciences, Tokushima City, Japan

* Corresponding author: Sachiko Chikahisa, Ph.D.

Department of Integrative Physiology, Tokushima University Graduate School of Biomedical Sciences, 3-18-15 Kuramoto-cho, Tokushima, 770-8504, Japan.

E-mail address: chika@tokushima-u.ac.jp, Tel: +81 88 633 7057, Fax: +81 88 633 9251

**
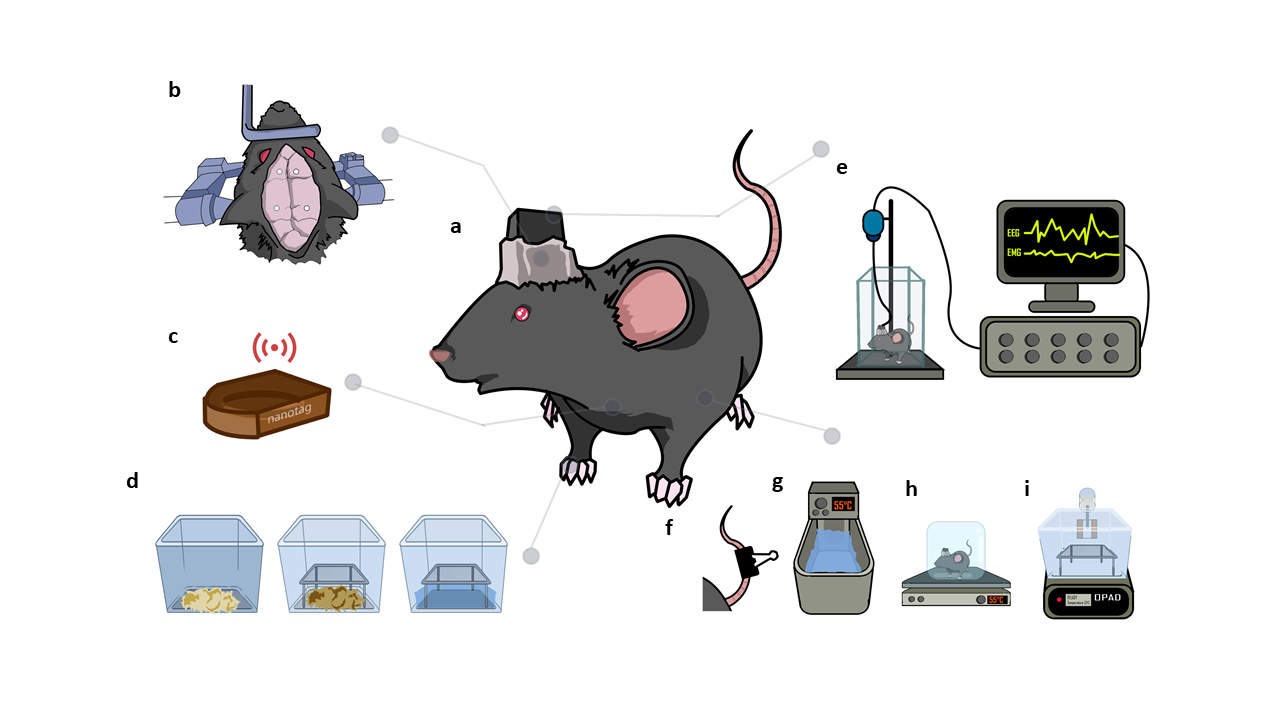
**

**Supplemental Figure S1. Sleep analysis and quantitative sensory testing for pain via a continuous model of PCMS.** Experiments were done on C57BL6/J male mice **(a)**. Stereotactic implantation of electroencephalograph and electromyograph electrodes was done before the start of the predictable chronic mild stress (PCMS) **(b)**. Telemetry device for the recording of locomotor activity and core body temperature was implanted in the peritoneal cavity **(c)**. Experimental cohorts consisted of a control, mesh wire, and water **(d)**. The vigilance states were ascertained through polysomnographic recording and pattern analysis **(e)**. Tail clip test was performed for mechanical hyperalgesia **(f)**. Tail immersion test was implemented for thermal hyperalgesia **(g)**. Hot plate test was used to measure frequency and reaction time to thermal stimulus **(h)**. The orofacial pain assessment device was used to ascertain orofacial pain **(i)**.

**
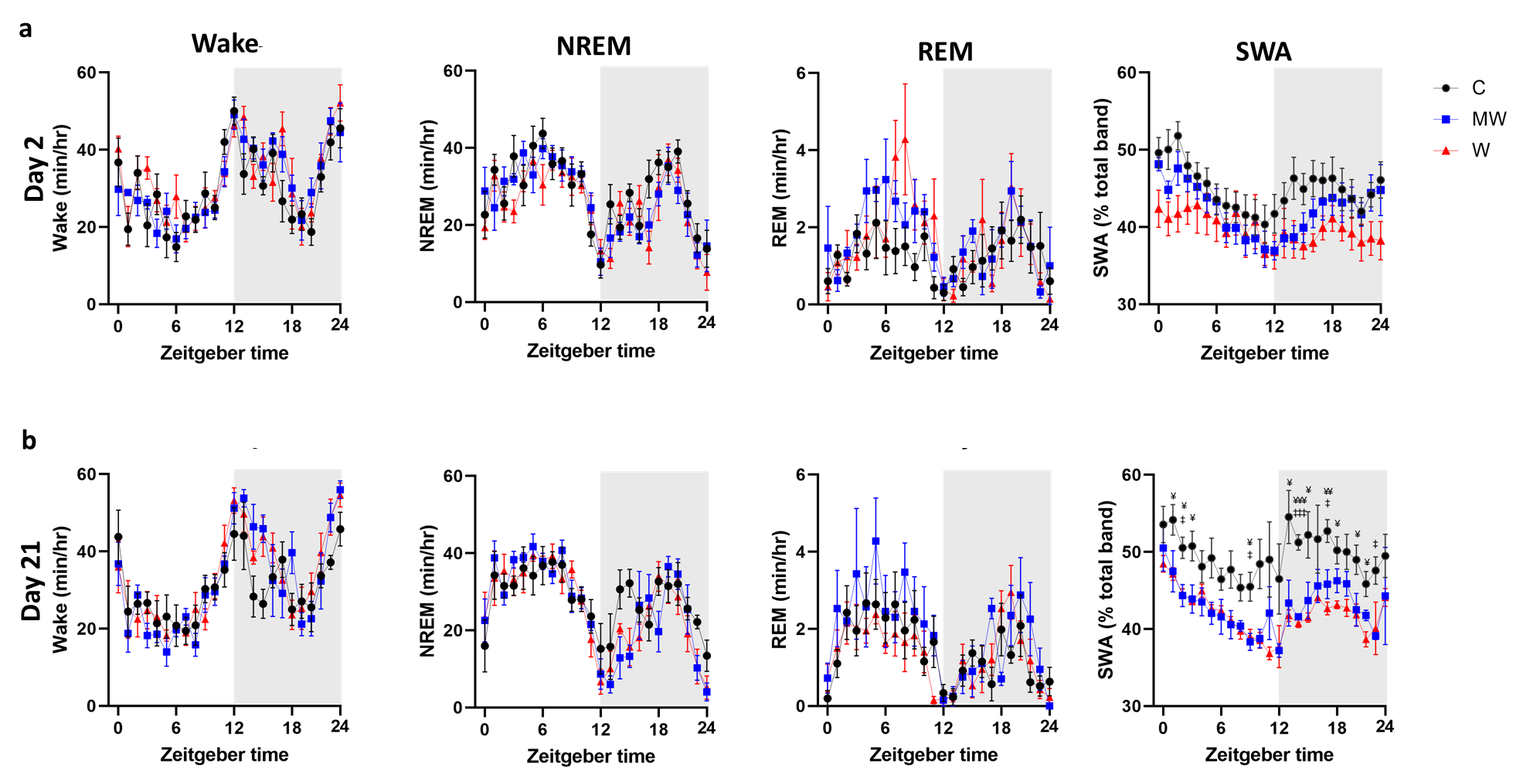
**

**Supplemental Figure S2. Hourly sleep-wake stages during days 2 and 21 of the PCMS timeline.** On day 2, predictable chronic mild stress (PCMS) caused no conspicuous variations in the hourly amount of wake, non-rapid eye movement (NREM) sleep, rapid eye movement (REM) sleep, and slow-wave activity (SWA) during NREM in the PCMS groups compared to the control (C) group **(a)**. On day 21, PCMS significantly decreased SWA during NREM sleep **(b)**. N=4-6 per group; ‡p<0.05 (mesh wire (MW) vs C); two-way repeated-measures ANOVA followed by Dunnett’s multiple comparison test. Data are presented as means ± SEM.

**
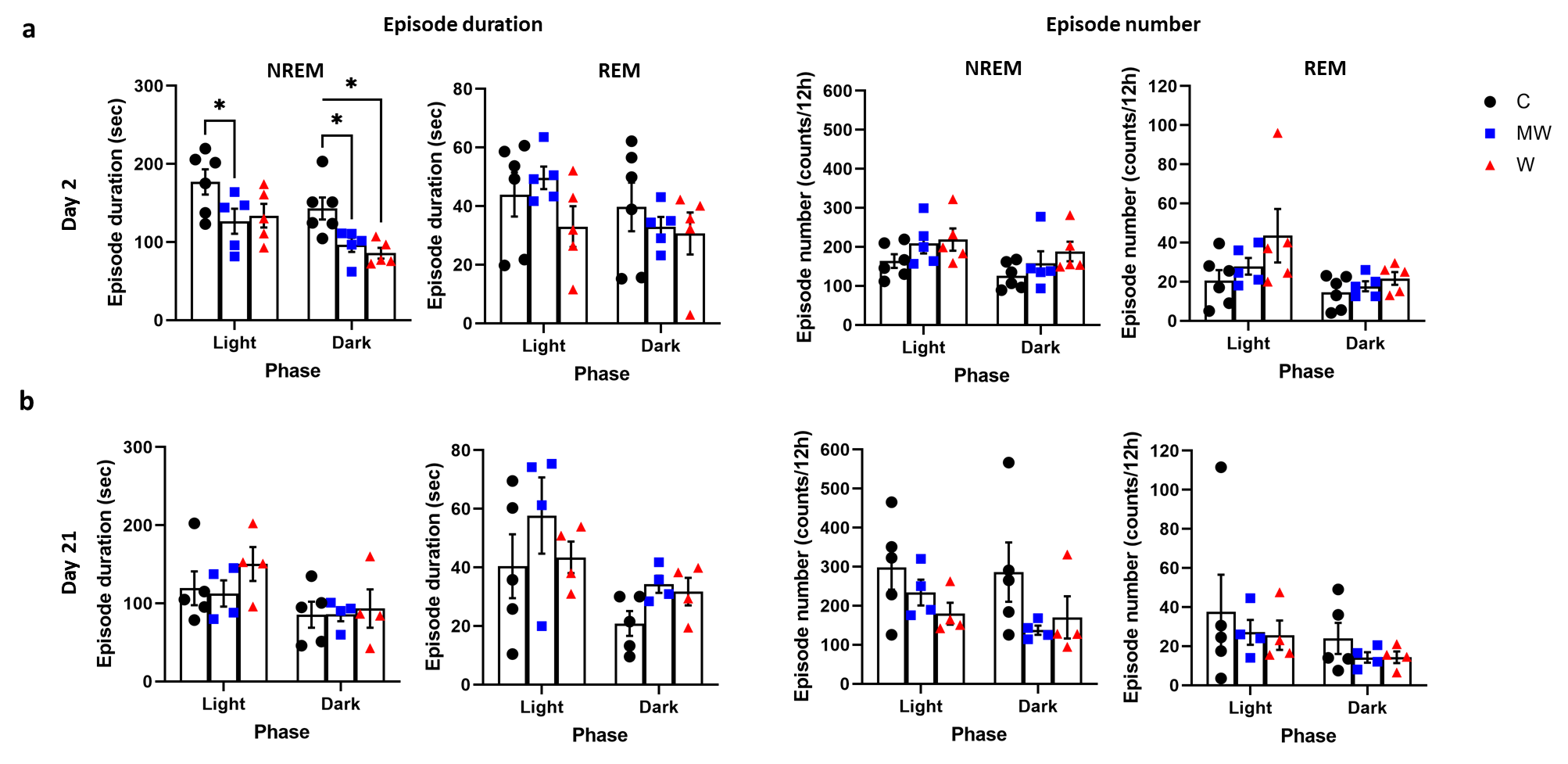
**

**Supplemental Figure S3. Episode duration and number of NREM sleep and REM sleep under PCMS.** On day 2 of predictable chronic mild stress (PCMS), episode duration of non-rapid eye movement (NREM) sleep decreased **(a)**. On day 21, episode number of NREM sleep tended to decrease in mesh wire (MW) and water (W) group during the dark phase compared to the control (C) **(b)**. *p<0.05, **p<0.01; two-way ordinary ANOVA followed by Dunnett’s multiple comparison test. Data are presented as means ± SEM.

**
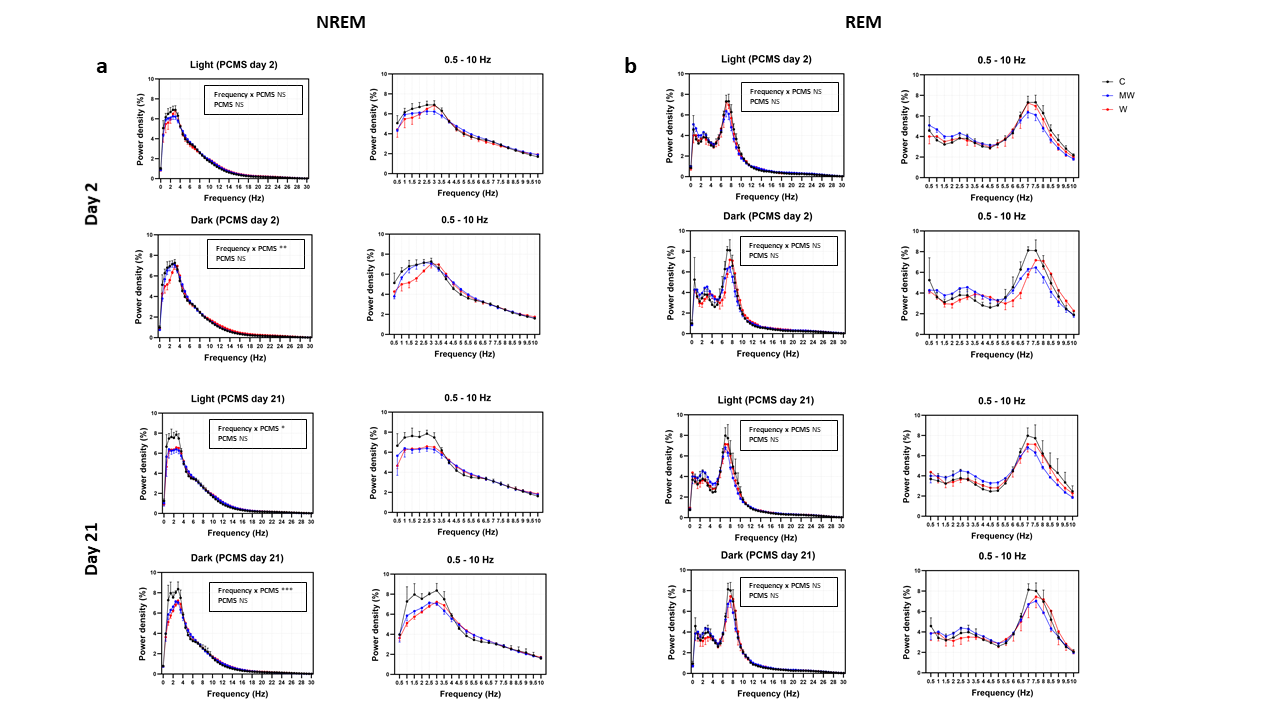
**

**Supplemental Figure S4. PCMS decreased power spectra during NREM sleep.** Electroencephalograph (EEG) power spectra during non-rapid eye movement (NREM) sleep on days 2 and 21 during the light and dark phases **(a)**. EEG power spectra at rapid eye movement (REM) sleep on days 2 and 21 during the light and dark phases **(b)**. two-way repeated-measures ANOVA followed. Data are presented as means ± SEM.
